# A PAI‐1 antagonist ameliorates hypophosphatemia in the *Hyp* vitamin D‐resistant rickets model mouse

**DOI:** 10.1002/2211-5463.13745

**Published:** 2023-12-25

**Authors:** Cheng Qian, Nobuaki Ito, Kunikazu Tsuji, Shingo Sato, Katsushi Kikuchi, Toshitaka Yoshii, Toshio Miyata, Yoshinori Asou

**Affiliations:** ^1^ Department of Orthopedics Surgery Tokyo Medical and Dental University Japan; ^2^ Division of Nephrology and Endocrinology The University of Tokyo Hospital Japan; ^3^ United Centers for Advanced Research and Translational Medicine Tohoku University Sendai Japan; ^4^ China‐Japan Friendship Institution of Medicine Shanghai University China

**Keywords:** fibroblast growth factor 23, *Hyp* mouse, hypophosphatemia, plasminogen activator inhibitor‐1 antagonist, vitamin D‐resistant rickets and osteomalacia

## Abstract

Congenital fibroblast growth factor 23 (FGF23)‐related hypophosphatemic rickets/osteomalacia is a rare bone metabolism disorder characterized by hypophosphatemia and caused by genetic abnormalities that result in excessive secretion of FGF23. *Hyp* mice are a model of X‐linked hypophosphatemia (XLH) caused by deletion of the *PHEX* gene and excessive production of FGF23. The purpose of this study was to investigate the potential of TM5614 as a therapeutic agent for the treatment of congenital FGF23‐related hypophosphatemic rickets and osteomalacia in humans by administering TM5614 to *Hyp* mice and examining its curative effect on hypophosphatemia. After a single oral administration of TM5614 10 mg·kg^−1^ to female *Hyp* mice starting at 17 weeks of age, the serum phosphate concentration increased with a peak at 6 h after administration. ELISA confirmed that TM5614 administration decreased the intact FGF23 concentration in the blood. Expression of 25‐hydroxyvitamin D‐1α‐hydroxylase protein encoded by *Cyp27b1* mRNA in the kidney was suppressed in *Hyp* mice, and treatment with 10 mg·kg^−1^ of TM5614 normalized the expression of 25‐hydroxyvitamin D‐1α‐hydroxylase protein and *Cyp27b1* mRNA in the kidneys of these mice. Our data indicate that oral administration of TM5614 ameliorates hypophosphatemia in *Hyp* mice, suggesting that TM5614 may be an effective treatment for congenital FGF23‐related hypophosphatemic rickets and osteomalacia.

## Graphical abstract

Plasminogen activator inhibitor‐1 (PAI‐1) antagonists activate tPA and Furin to degrade fibroblast growth factor 23 (FGF23). We examined TM5614, a PAI‐1 antagonist, as a potential therapeutic agent for FGF23‐associated hypophosphatemic rickets. Oral administration of TM5614 to Hyp mice, an X‐linked hypophosphatemia (XLH) model, caused normalization of serum phosphate levels as intact FGF23 levels decreased. TM5614 may be a therapeutic candidate for FGF23‐associated hypophosphatemic rickets.

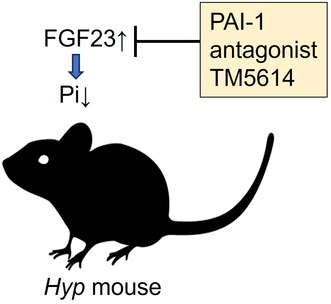

**Abbreviations**
Cyp24a1: cytochrome P450 Family 24 Subfamily A Member 1
Cyp27b1: cytochrome P450 Family 27 Subfamily B Member 1
ELISA: enzyme‐linked immunosorbent assay
FGF23: fibroblast growth factor 23
*Hyp*
: X‐linked hypophosphatemic
IHC: immunohistochemistry
*kl*
: *klotho*
NaPi2A/2C: Na^+^‐Pi cotransporter 2a/2C
PA: plasminogen activator
PAI‐1: plasminogen activator inhibitor‐1
PHEX: phosphate‐regulating gene with homologies to endopeptidases on the X chromosome
XLH: X‐linked hypophosphatemia

Rickets is a bone calcification disorder caused by abnormal calcium and phosphorus metabolism. The condition in childhood that develops before the closure of the epiphyseal line is called rickets, while the condition after the closure of the epiphyseal line is called osteomalacia. Rickets/osteomalacia is classified into vitamin D deficiency/dependent rickets/osteomalacia and vitamin D‐resistant rickets/osteomalacia. The latter includes fibroblast growth factor 23 (FGF23)‐related hypophosphatemic rickets/osteomalacia, Fanconi syndrome, and hereditary hypophosphatemic rickets with hypercalciuria [1]. Congenital FGF23‐related hypophosphatemic rickets/osteomalacia is a rare bone metabolism disorder characterized by hypophosphatemia due to excessive secretion of FGF23 caused by genetic abnormalities. The estimated number of new cases of FGF23‐related hypophosphatemic rickets and osteomalacia per year in Japan is 117 [2]. Most cases of vitamin D‐resistant osteomalacia are caused by overproduction of FGF23, a hormone that promotes urinary excretion of phosphate, resulting in low serum phosphate levels and impaired bone calcification [3, 4].

Belonging to a subfamily of endocrine FGFs, FGF23 is a phosphaturic hormone [5]. It is secreted by osteoblasts and osteocytes into the systemic circulation and exerts its effects in the kidney, parathyroid, heart, bone, and potentially other organs [6]. X‐linked hypophosphatemia (XLH) is the most commonly inherited form of rickets among humans, caused by inactivating mutations in the phosphate‐regulating gene with homologies to endopeptidases on the X chromosome (*PHEX*) [7, 8, 9]. In the same fashion, a loss of function variant in the murine homolog of PHEX, brings about an XLH‐like characteristic in *Hyp* mice, a renowned animal model for XLH [10, 11, 12]. For individuals with XLH and *Hyp* mice, the typical result of abnormally elevated FGF23 levels is an increase in renal phosphate excretion and subsequent hypophosphatemia due to the reduced expression of sodium‐dependent phosphate transporters in the proximal tubule 2A and 2C (NaPi2A/2C), as well as a decrease in 1,25D levels through the suppression of renal 1‐α hydroxylase (encoded by *Cyp27b1*) and the activation of the catabolic 24‐hydroxylase (encoded by *Cyp24a1*) [13].

Plasminogen activator inhibitor‐1 (PAI‐1) is a serine protease inhibitor (SERPIN) that inhibits the activity of plasminogen activator (PA) and Furin [14, 15, 16]. Recently, it has been shown that administration of PAI‐1 antagonist or genetic PAI‐1 deficiency suppresses serum phosphate levels in mice apparently by the FGF23 protein degradation by PA and Furin, thereby suppressing serum phosphate levels [14, 17]. These observations indicate that PAI‐1 plays an important role in FGF23 homeostasis.

TM5614, a novel small‐molecule antagonist of PAI‐1, was obtained as a lead compound from TM5007, which was obtained based on the X‐ray crystal structure information of PAI‐1 [18, 19, 20]. TM5614 is the orally available small‐molecule PAI‐1 antagonist and its safety in humans has been confirmed in clinical trials [21].

Based on the findings above, we decided to administer TM5614 to the *Hyp* mice to verify whether TM5614 can reduce the total amount of circulating intact FGF23 in the *Hyp* mouse and thus whether it can increase the serum phosphate concentration of the *Hyp* mouse and enhance the expression of *Cyp27b1*.

## Materials and methods

### Animals

All animal experiments were approved by the Animal Care and Use Committee of Tokyo Medical and Dental University and were carried out in accordance with the approved guidelines (approval number: A2018316). Female *Hyp* mice (B6.Cg‐Phex^*Hyp*^/J) and male wild‐type (WT) were purchased from Jackson Laboratory (Bar Harbor, ME, USA). To obtain *hyp*/+ and +/+ females for use in the experiment, *hyp*/+ females and +/y males were crossed. Mice were caged in groups of 3–5, maintained on a 12‐h dark/light cycle, were provided standard rodent food pellets (Labo MR Stock, Nosan, Tokyo, Japan), and sterilized water. We used the following sequences for genotyping to determine the mouse genotype. Forward of both WT and Mutant: 19794 TCC AAA GCT GTC TGA AAC TCC, WT Reverse: TCT GCT TAA GTG GCC CAA TG, and Mutant Reverse: TAG CTC TGA ACC TCA GTT TCT TCA.

### Single administration

Female *Hyp* or WT mice were administrated with TM5614 in a form of suspension at a dosage of 10 mg·kg^−1^ body weight by orally when they were 17 weeks old (*n* = 10 for Hyp mice, *n* = 8 for WT mice). The dosage of TM5614 was determined with reference to previous studies [14]. They administered various doses of TM5441, a derivative of TM5614, to klotho mice overexpressing FGF23 and found that 10 mg·kg^−1^ was optimal. Preparation method of TM5614 suspension was grinded TM5614 powder into suspension with 5% vehicle (CMC‐Na) and saline solution. The suspension of the vehicle was given orally as indicated below. Blood was taken from the retro‐orbital sinus before administration (once) and after administration by the following time points: 3, 6, 9, and 12 h by a blood‐collecting vessel (heparinized; Hirschmann, Eberstadt, Germany). We ensured that each administration was given at 9 a.m., and blood was collected at 12 p.m., 3 p.m., 6 p.m., and 9 p.m. The blood samples were centrifuged at 9000 ***g*** for 6 min to obtain serum. All samples are stored at −80 °C to be used for phosphate concentration measurement.

### Continuous administration

We administered TM5614 to *Hyp* or WT mice orally at an age of 17 weeks at a dose of 10 mg·kg^−1^ every day for 10 days (*n* = 10 for Hyp mice, *n* = 8 for WT mice). Blood was taken from the retro‐orbital sinus at the following time point: before administration, 6 h after administration on the 1st day, 2nd day, 4th day, 6th day, 8th day, and 10th day. We ensured that each administration was given at 9 a.m. every day until the 10th day, and blood was collected at 3 p.m. at the time point mentioned above. The blood samples were centrifuged at 9000 ***g*** for 6 min to obtain serum. All samples are stored at −80 °C to be used for phosphate concentration measurement.

### Serum phosphate concentration

Serum phosphate concentration was tested by an automated clinical chemistry analyzer (DRI‐CHEM NX500, Fujifilm, Tokyo, Japan).

### Serum FGF23 concentration

Serum levels of FGF23 (intact and c‐term FGF23) were quantified using mouse intact and c‐term ELISA kit (Quidel, CA, USA) [13].

### RNA isolation, RT‐PCR, and qPCR analysis

Total RNA of femur cortical bone and kidney isolated using TRIzol reagent (Invitrogen, Burlington, ON, Canada) was quantified by spectrophotometric readings at 260/280 nm. Total RNA (1 μg) was reverse transcribed (Super Script VILO cDNA Synthesis Kit; Invitrogen) and used for determining the expression of *Fgf23* (in the femur) and *Cyp27b1* (in the kidney). Mouse‐specific primers were designed using the Primer Express software, version 3.0 (Applied Biosystems, Foster City, CA, USA). The following primers were used for Fgf23: FGF23 forward 5′‐ATGCTTCTGCGACAAGTAGAC‐3′ and FGF23 reverse 5′‐TGACTCGAAGGTTCCTTTGTATG‐3′. And primers for Cyp27b1, CYP27B1 forward 5′‐CTCTGGGCAAAGGCAAACATCTGA‐3′, and CYP27B1 reverse 5′‐CCGCGGGCTATGCTGGAAC‐3′ were used. Polymerase chain reaction (PCR) was performed on a StratageneMx3000p System (Agilent Technologies Japan, Ltd., Hachioji‐shi, Tokyo, Japan) by using the Kapa SYBR Fast qPCR Kit (Kapa Biosystems, Inc., Boston, MA, USA). The expression of mRNAs was normalized to that of β‐actin, and fold differences were calculated using the ΔΔ*C*_t_ method [22].

### Immunohistochemistry (IHC)

Kidneys were removed by dissection and fixed in 4% paraformaldehyde. After dehydration and paraffin embedding, serial 5‐μm‐thick sagittal sections were made. Kidney paraffin sections were deparaffinized and dehydrated. Three percent hydrogen peroxide in methanol was used to block endogenous peroxidase activity. All sections were afterward incubated with 5% goat serum to minimize cross‐reactivity. Sections were incubated with recombinant anti‐Cyp27b1 antibody (dilution 1 : 2000, EPR20271, Abcam, Cambridge, UK) overnight. Next, sections were incubated with secondary antibody Goat Anti‐Rabbit IgG H&L (HRP; dilution 1 : 500, ab205718, Abcam) for 20 min at room temperature. Signals were visualized by peroxidase‐conjugated avidin and diaminobenzidine using a Vectastain ABC kit (Vector Laboratories, Newark, CA, USA). Negative control sections were incubated with T‐PBS solution instead of anti‐Cyp27b1 antibody [23]. The positive stained cells were counted and normalized per tissue area. Quantification of immune‐positive cells was performed by three independent raters. There was no predominant inter‐rater variability in the results of the three evaluations.

### Statistical analysis

Data were expressed as mean ± standard deviation (SD), and Student's *t*‐test, paired *t*‐test, and two‐way ANOVA, when necessary, were applied to analyze the differences among groups. In all statistical analyses, the significance was set for *P*‐values <0.05. Statistical analyses were performed using the graphpad prism 9 software (GraphPad Software, Boston, MA, USA).

## Results

### Single TM5614 administration

#### TM5614 administration to *Hyp* mice increased phosphate levels with a peak 6 h after administration

**Fig. 1:**
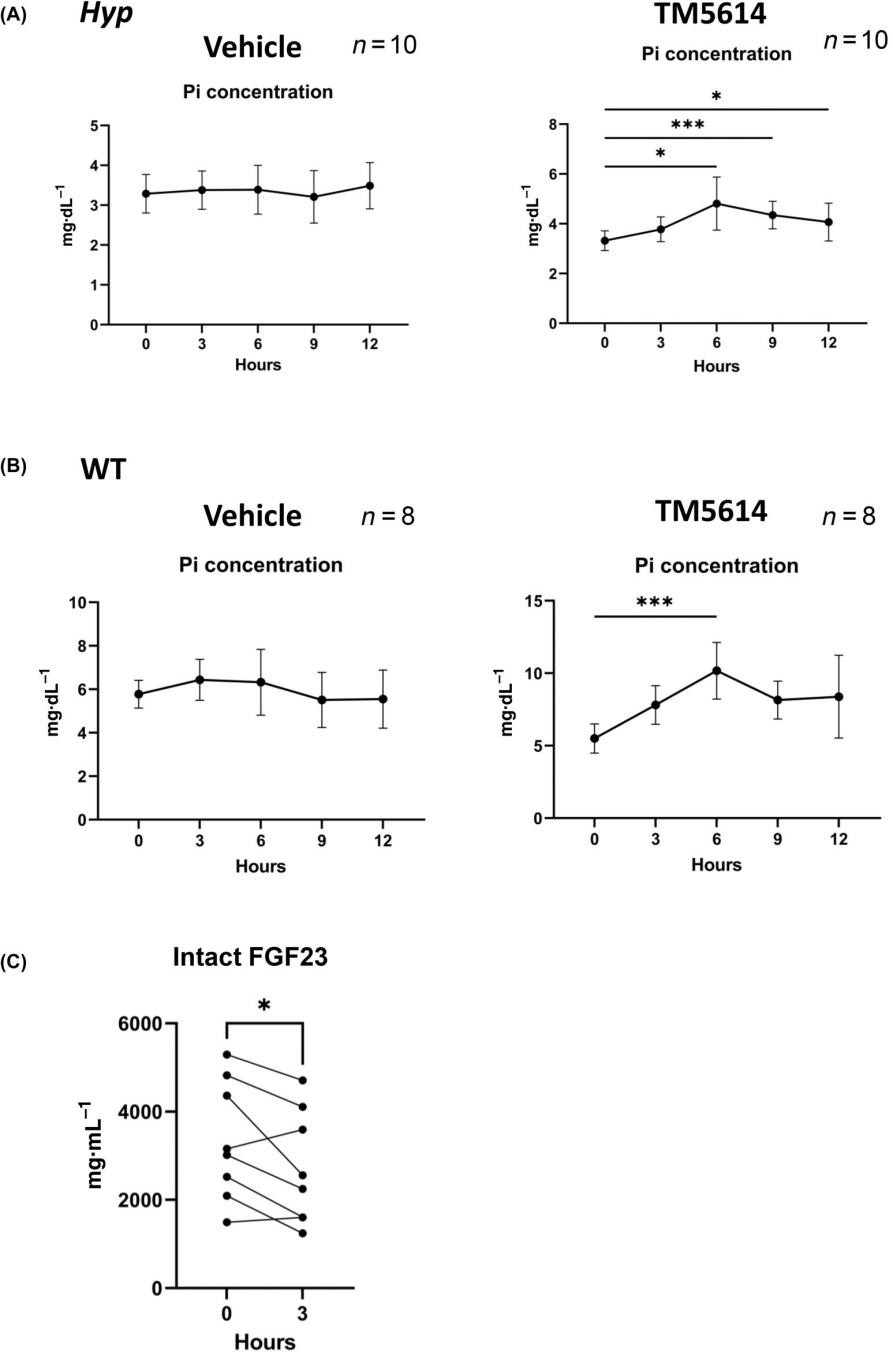
Single dose of TM5614 increases serum phosphate levels. Phosphate concentration after single administration of vehicle (left) or TM5614 (right)in *Hyp* mice (A) or wild‐type (WT) mice (B) (*n* = 10). (C) Serum intact fibroblast growth factor 23 (FGF23) concentration in *Hyp* mice before and 3 h after administration (*n* = 8). Data are presented as the mean ± standard deviation. Results from the paired *t*‐test are presented in the figure legends. *P < 0.05 and ***P < 0.001 versus untreated. Those without pairwise comparisons stand for no significance.

TM5614 (10 mg·kg^−1^ body weight) was administered orally, and blood samples were taken from the posterior orbital sinus before and at 6, 9, and 12 h after administration to evaluate changes in serum phosphate levels. As a result, serum phosphate levels in *Hyp* mice and in WT mice peaked at 6 h after administration (Fig. 1A,B). In contrast, no changes in serum phosphate levels were observed in *Hyp* mice or WT mice in the Vehicle treatment group. Next, the effect of TM5614 on serum levels of intact FGF23 protein in *Hyp* mice was examined. The results showed that the concentration of intact FGF23 protein in serum significantly decreased at 3 h after TM5614 administration, prior to the peak of phosphorus concentration (Fig. 1C).

#### Continuous daily TM5614 administration to *Hyp* mice persistently evaluated blood phosphate levels without effect on *Fgf23* mRNA in the femur

**Fig. 2:**
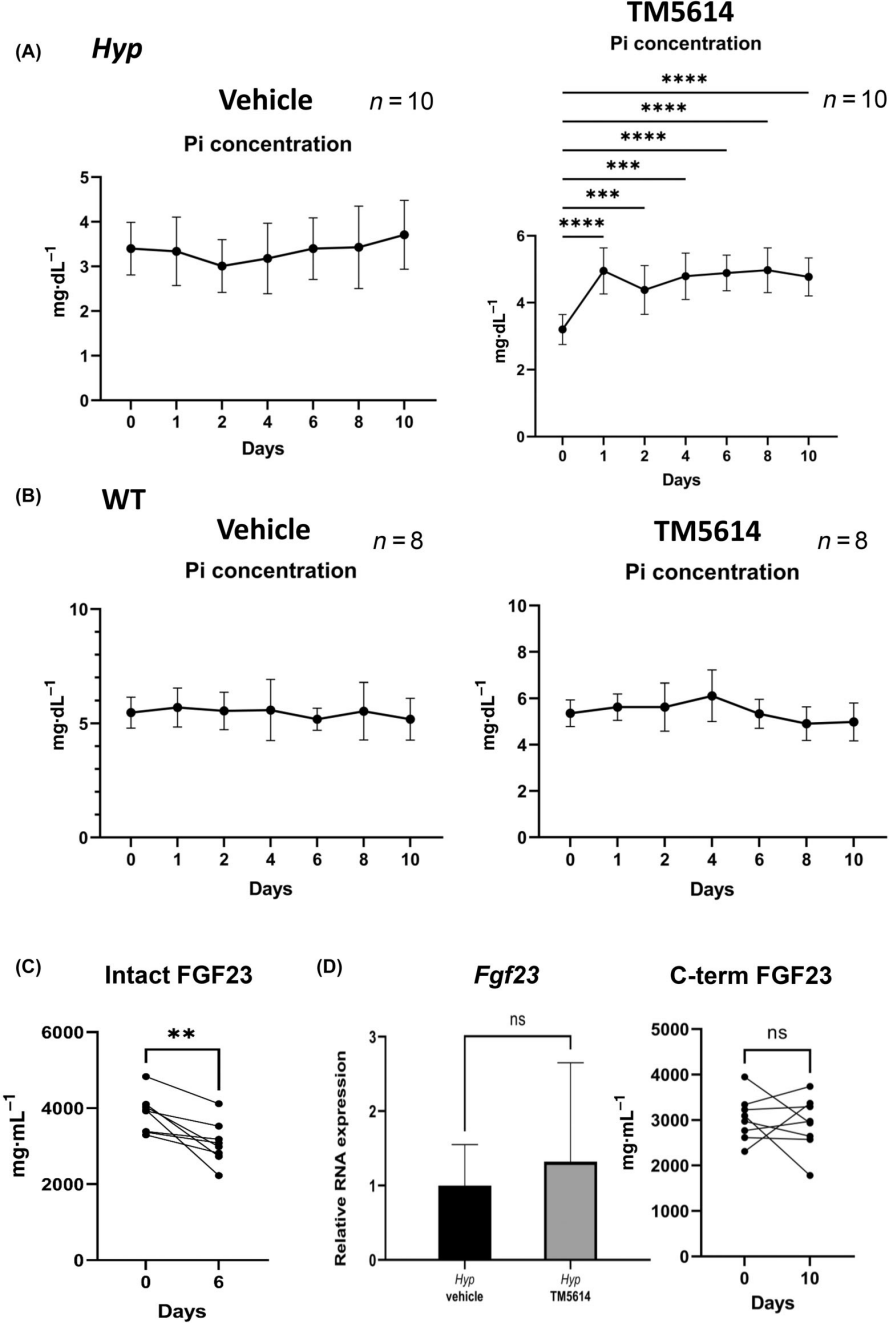
Daily administration of TM5614 increases serum phosphate levels in *Hyp* mice. Phosphate concentration after continuous daily TM5614 administration of vehicle (left) or TM5614 (right) in *Hyp* mice (A) and wild‐type mice (B). (C) Serum intact fibroblast growth factor 23 (FGF23) concentration in *Hyp* mice before and after 6 days of TM5614 administration (*n* = 8). (D) The expression of mRNA *Fgf23* (*n* = 6) and C‐term Fgf23 protein (*n* = 7) of in *Hyp* mouse after continuous daily TM5614 or vehicle administration. Data are presented as the mean ± standard deviation. Results from the paired *t*‐test are presented in the figure legends. **P < 0.01, ***P < 0.001 and ****P < 0.0001 versus untreated. Those without pairwise comparisons stand for no significance.

Based on the results of the single administration experiment, TM5614 was administered once daily for 10 consecutive days. On Days 1, 2, 4, 6, 8, and 10 after dosing, blood samples were taken at 6 h postadministration to measure serum phosphate levels, respectively. The results showed that serum phosphate levels were consistently elevated in *Hyp* mice (Fig. 2A). On the contrary, in WT mice and in the vehicle group, the serum phosphate concentration did not increase at any time point compared with that before administration (Fig. 2B). The concentration of intact FGF23 protein in serum significantly decreased at 6 days of TM5614 administration (Fig. 2C). Next, to determine whether TM5614 affects *Fgf23* transcription, we performed qPCR analysis of femur samples from *Hyp* mice in the daily administration group. The results showed that daily administration of TM5614 had no significant effect on *Fgf23* mRNA in the femur (Fig. 2D). At this time point, the concentration of c‐term FGF23 protein in serum was also similar between the TM5614 administration group and the vehicle group (Fig. 2D).

#### Continuous daily TM5614 administration enhanced the expression of *Cyp27b1* mRNA and 25‐hydroxyvitamin D‐1α‐hydroxylase protein

**Fig. 3:**
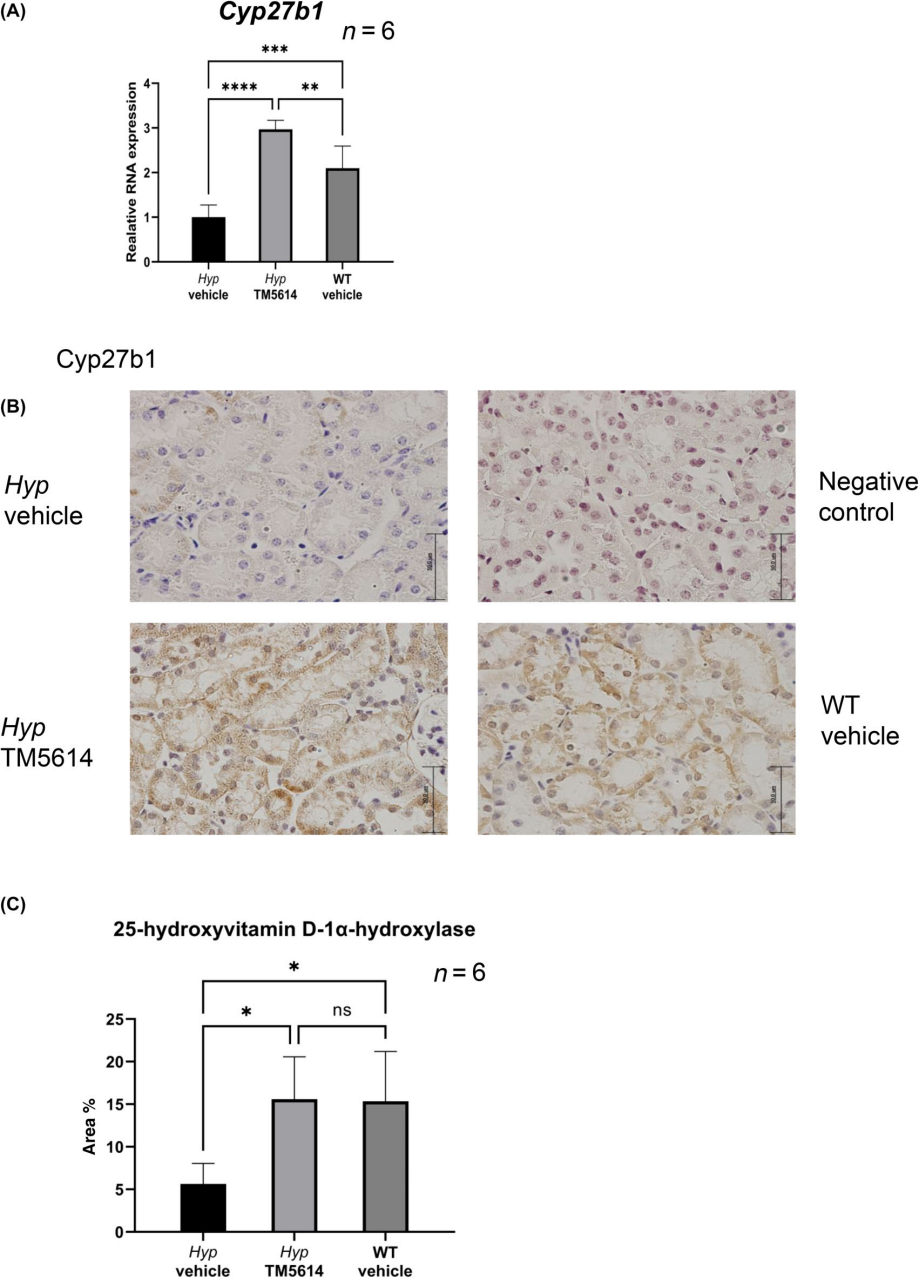
TM5614 promotes expression of 25‐hydroxyvitamin D‐1α‐hydroxylase in *Hyp* mice. (A) Kidney gene expression of *Cyp27b1* in *Hyp* mice or wild‐type (WT) mice after continuous daily administration of TM5614 or vehicle (*n* = 6). (B) Representative IHC images of 25‐hydroxyvitamin D‐1α‐hydroxylase in the kidneys of *Hyp* mice and WT mouse (scale bar = 50 μm). (C) Proportion of 25‐hydoroxybitmian D‐1αhydroxylase positive area in kidney sections (*n* = 6). Data are presented as the mean ± standard deviation. Results from the *t*‐test are presented in the figure legends. *P < 0.05, **P < 0.05, ***P < 0.001 and ****P < 0.0001 versus untreated, ns, not significant.

FGF23 suppresses vitamin D activation in the proximal tubule via regulation of *Cyp27b1* expression, which encodes 25‐hydroxyvitamin D‐1α‐hydroxylase [13]. Based on this finding, we evaluated the effect of TM5614 treatment on *Cyp27b1* expression in the kidney. Kidneys were harvested from *Hyp* mice treated with TM5614 or vehicle for 10 consecutive days, and WT mice with vehicle for 10 consecutive days. The kidneys were analyzed for *Cyp27b1* mRNA expression by qPCR. In the vehicle‐treated group, *Cyp27b1* mRNA expression was decreased in the kidneys of the vehicle‐treated *Hyp* mice compared with WT mice. On the contrary, *Cyp27b1* mRNA expression in *Hyp* mice treated with TM5614 was significantly elevated compared with the vehicle‐treated *Hyp* mice and exceeded the expression levels in vehicle‐treated WT mice (Fig. 3A).

Immunohistochemical staining revealed that the expression of 25‐hydroxyvitamin D‐1α‐hydroxylase protein in the kidneys of vehicle‐treated *Hyp* mice was also decreased compared with WT mice, while it was enhanced to almost the same levels as WT mice level in the TM5614‐treated *Hyp* mice (Fig. 3B,C).

## Discussion

In this study, we investigated the potential of TM5614, a novel molecular antagonist of PAI‐1, to ameliorate hypophosphatemia in *Hyp* mice. The results showed that administration of TM5614 decreased intact FGF23 levels, which in turn increased phosphate levels and promoted the expression of *Cyp27b1*, an enzyme that regulates vitamin D activation *in vivo*. These results suggest that TM5614 could be used as a therapeutic agent for vitamin D‐resistant rickets/osteomalacia caused by excessive FGF23 secretion.

PAI‐1 antagonists activate tPA and uPA [14, 15, 16] and both promote direct FGF23 cleavage by themselves extracellularly, while intracellularly they promote FGF23 cleavage by Furin activation and reduce serum intact FGF23 levels [17]. *In vitro* experiments showed that tPA was more active than uPA and cleaved FGF23 after 1 h of treatment [17]. In the present study, serum intact FGF23 levels decreased at 3 h after TM5614 administration, prior to the increase in phosphate levels, which peaked at 6 h after administration, consistent with the above results.

A single dose of TM5614 increased serum phosphate levels in both *Hyp* mice and WT mice, while TM5614 daily administration increased serum phosphate levels only in *Hyp* mice. The reason for this is not clear, but it may be that *Hyp* mice have higher FGF23 levels and the effect of TM5614, which degrades FGF23, is more easily seen, whereas WT mice have lower FGF23 levels and the effect of daily administration was less apparent.

In the present study, we show that TM5614 promotes the expression of *Cyp27b1* mRNA and 25‐hydroxyvitamin D‐1α‐hydroxylase protein in the kidney in response to decreased FGF23 levels. In recent years, the functions of FGF23 outside of phosphate metabolism have become increasingly clear. For example, in the heart, FGF23 might cause left ventricular hypertrophy in a *klotho‐*independent manner via FGFR4 [6]. In addition, the premature aging phenotype exhibited by *kl/kl* mice is partially rescued by suppression of FGF23 through PAI‐1 inhibition, and yet the lifespan is extended [14]. This fact suggests that FGF23 may be one of the regulators of aging; suppression of FGF23 by TM5614 may also affect these signals and regulate biological homeostasis.

Recently, burosumab, a fully humanized monoclonal antibody against FGF23, was listed in the NHI drug price list in Japan for the indication of FGF23‐related hypophosphatemic rickets and osteomalacia [24, 25]. Compared with the conventional treatment with a combination of phosphate and active vitamin D preparations, burosumab, which targets the main symptom of the disease, elevated FGF23 in the serum, has been reported to have improved therapeutic results. However, adherence is a concern for patients with FGF23‐related hypophosphatemic rickets/osteomalacia who require long‐term treatment, as the drug requires subcutaneous injection once a month in adults and twice a month in children. In addition, from a healthcare economic point of view, burosumab treatment, an antibody agent, must be expensive. In this respect, TM5614, which can be taken orally, has an advantage.

Although it is possible that increased FGF23 transcription could occur as a positive feedback after TM5614 treatment caused a decrease in intact FGF23 concentration followed by an increase in phosphate concentration, in the present study, FGF23 transcription in bone tissue was not increased even after TM5614 treatment. Although the function of PAI‐1 on FGF23 transcription is unknown, we have recently shown that PAI‐1‐deficient mice have decreased FGF23 mRNA expression in bone tissue [26]. PAI‐1 inhibition prevents cell senescence, but promotes FGF23 transcription in senescent cells [26]. It is not known whether the senescence of osteocytes is enhanced in XLH mice, and the molecular mechanism of FGF23 transcription is an area for future study.

The limitation of this experiment is that the effect of TM5614 on bone metabolism could not be verified due to the short duration of TM5614 administration. To verify the effect of TM5614 on bone metabolism, it is necessary to administer TM5614 for several months, but this could not be done in this study because it is difficult to administer TM5614 by gavage for a long period of time. However, other studies found that sclerostin antibody treatment improved *Hyp* mouse bone mass, bone length, and decreased intact FGF23 levels [27], and calcitriol was able to normalize *Hyp* mouse body weight and femur length [13, 28, 29]. Additionally, burosumab, a monoclonal antibody against the FGF23 protein, increased bone length in *Hyp* mice [30] and may provide another potential option for combination therapy with TM5614. In addition, since the results obtained in this study were the effects of administration to mice, the possibility cannot be denied that the effects of administration to humans may be different. Although TM5614 treatment promoted *Cyp27b1* expression, it remains unclear whether it had an effect on vitamin D activation, since it is difficult to measure 1, 25‐(OH)2 Vitamin D in mice and the effect on Cyp24a1, an important enzyme in vitamin D inactivation, has not been analyzed. Further *in vivo* experiments are needed for these analyses.

This study demonstrates that the PAI‐1 antagonist TM5614 may be an effective treatment for vitamin D‐resistant rickets and osteomalacia caused by excessive production of FGF23. TM5614 is in advanced human clinical trials and has already completed safety studies [21]. TM5614 is expected to be developed as a therapeutic agent with good adherence for patients because it is a low molecular weight compound that can be produced inexpensively and is an orally available drug.

## Conflict of interest

TM declares research funding from Astellas, Daiichi Sankyo, Kowa, and stocks of Renascience. CQ, NI, KT, SS, TY, and YA declare that they have no conflict of interest.

### Peer review

The peer review history for this article is available at https://www.webofscience.com/api/gateway/wos/peer‐review/10.1002/2211‐5463.13745.

## Author contributions

YA, NI, and TM contributed to the conception. CQ, SS, and KT contributed to the interpretation or analysis of data. CQ, YA, and NI contributed to the preparation of the manuscript. YA, TM, and TY contributed to the supervision. YA and NI designed the study. CQ, SS, and KT contributed to the animal studies and statistical analyses. SS, TY, KK, and TM contributed reagents, materials, and analysis tools. YA and NI critically revised the manuscript. All authors approved the final manuscript.

## Data Availability

All the data generated or analyzed during this study are included in the published article.
